# Fabry disease in W162C mutation: a case report of two patients and a review of literature

**DOI:** 10.1186/s12883-024-03540-3

**Published:** 2024-04-05

**Authors:** Alessandro Furia, Raffaello Ditaranto, Elena Biagini, Vanda Parisi, Alex Incensi, Sara Parisini, Rocco Liguori, Vincenzo Donadio

**Affiliations:** 1 IRCCS Istituto delle Scienze Neurologiche di Bologna, UOC Clinica Neurologica, Via Altura 3, 40139 Bologna, Italy; 2 Cardiology Unit, IRCCS Azienda Ospedaliero-Universitaria di Bologna, Bologna, Italy; 3 European Reference Network for Rare, Low Prevalence, and Complex Diseases of the Heart (ERN GUARD-Heart), Bologna, Italy; 4 Dipartimento di Scienze Biomediche e Neuromotorie, Università di Bologna, Bologna, Italy

**Keywords:** Fabry disease, Heart, Skin, Biopsy, Globotriaosylceramide

## Abstract

**Background:**

Fabry disease is a multisystemic disorder characterized by deposition of globotriaosylceramide (Gb3) and its deacylated form in multiple organs, sometimes localized in specific systems such as the nervous or cardiovascular system. As disease-modifying therapies are now available, early diagnosis is paramount to improving life quality and clinical outcomes. Despite the widespread use of non-invasive techniques for assessing organ damage, such as cardiac magnetic resonance imaging (MRI) for patients with cardiac disease, organ biopsy remains the gold standard to assess organ involvement.

**Case presentation:**

The cases of two patients, father and daughter with a W162C mutation, are described. The father presented with late-onset, cardiac Fabry disease, subsequently developing systolic dysfunction and heart failure. His daughter, while asymptomatic and with normal cardiac assessment (except for slightly reduced native T1 values by cardiac MRI), had already initial myocyte Gb3 deposits on the endomyocardial biopsy, allowing her to start therapy precociously and potentially modifying the course of her disease. A review of the literature concerning the W162C mutation is then provided, showing that it is usually associated to classic, multisystemic Fabry disease rather than the cardiac-restricted form as in these two cases.

**Conclusions:**

Three main points can be concluded from this report. First, the W162C mutation can present with a more variegate phenotype than that predicted on a molecular basis. Second, endomyocardial biopsy was shown in this case to precede non-invasive investigation in determining organ involvement, justifying further studies on this potentially reliable technique, Third, difficulties can arise in the management of asymptomatic female carriers.

## Background

Fabry disease (FD) is a lysosomal storage disorder caused by mutations of the α-galactosidase A gene (*GLA*) on the X chromosome, determining enzymatic deficiency and subsequent pathological deposition of globotriaosylceramide (Gb3) and its deacylated form globotriaosylsphingosine (lyso-Gb3) in cells of various tissues. While the classical form of FD, due to (almost) complete *GLA* deficiency, affects several organs and systems such as central and peripheral nervous system, skin, heart and kidneys, atypical forms are possible, where the disorder presents a later-onset involvement of a single or few organs (usually heart, in some cases kidneys). Moreover, while females were originally thought to be protected by the X-linked inheritance, disease spectrum may range from the status of asymptomatic carriers to a full-fledged disorder for those preferentially expressing disease-causing *GLA* mutation as a result of an unfavorable skewed X inactivation. Since disease-modifying treatments (i.e., enzyme replacement therapy (ERT) and oral chaperone for amenable mutations) are now available, early diagnosis has become a paramount issue.

 .

Here, we present the case of 2 relatives (father and daughter) with a later-onset FD mainly restricted to the heart carrying W162C mutation of the *GLA* gene. Due to the fact that the W162C mutation has been associated to classic FD and not to organ-restricted forms as in our two cases, a review of the literature concerning the mutation is provided.

## Case presentation

### First patient

The first patient was a 44-year-old man whose cardiological history started with the diagnosis of hypertrophic cardiomyopathy, which required dual chamber implantable cardioverter-defibrillator (ICD)CD implantation after development of severe atrioventricular block complicated by ventricular fibrillation. His family history was significant for cardiovascular disorders, as his mother passed away from stroke at 62 years of age. Review of past history showed arterial hypertension and prostatic hypertrophy, as well as scleroderma (however, this last diagnosis wasn’t confirmed buy subsequent clinical or laboratory data).

Two years later, the patient was admitted for an ischemic stroke and anticoagulation (warfarin) was started after evidence of paroxysmal atrial fibrillation. Serial cardiological assessment documented a progressive decline of left ventricle systolic function with an end-stage evolution over time. Subsequently, two episodes of ventricular tachycardia occurred, requiring transcatheter tissue ablation. At 69 years of age, urinalysis revealed proteinuria with normal serum creatinine. These clinical data led to suspicion of cardiac FD: genetic testing revealed a pathogenic missense mutation NM_000169.2: c.486G > T (p.W162C) in *GLA*. His lyso-GB3 levels were supportive of the diagnosis (73.13 ng/ml, normal values being < 2.7 ng/ml) and α-galactosidase A activity was undetectable (< 0.8 µmol/L/h [limit of detection]).

Cornea verticillata and angiokeratoma were not evident at dermatological and ophthalmological evaluation. From a neurological point of view, physical examination was unremarkable except for diffusely weak, but symmetric deep tendon reflexes. The patient reported an episode of migratory articular pain without definite paresthesia at 47 years of age, following a stressful event. At 56 years of age he suffered from transient monocular vision loss lasting several minutes, which was interpreted as a transient ischemic attack. A subsequent head CT scan demonstrated two ischemic lesions affecting the right thalamus and centrum semiovale. We performed skin biopsy searching for Gb3 accumulations in skin samples by an immunofluorescent indirect (IF) technique which were not found. ERT was started at 70 years of age without side effects.

Unfortunately, cardiomyopathy progress towards severe left ventricle systolic dysfunction with heart failure, as diagnosed from around 70 years of age. The patient remained clinically stable (New York Heart Association class IIb), until the age of 73 years when he was admitted due to acute-on-chronic heart failure. Long-term mechanical assistance with left ventricular assist device was deemed unfeasible by a multidisciplinary care team due to an unfavorable risk-benefit ratio. and the patient passed away a month after admission due to refractory heart failure.

### Second patient

The first patient’s daughter was identified through genetic family cascade screening (heterozygote for the familial *GLA* mutation), at the age of 22 and lyso-GB3 levels were slightly higher than normal (3 ng/ml). Her physical examination and personal history were unremarkable, apart from recurrent frontal headache episodes. Cardiac (12-lead ECG, 24 h-ECG Holter, echocardiogram, troponin, BNP) and renal (serum creatinine, 24 h proteinuria) evaluation were normal.

Neurological examination and brain MRI were negative. Skin biopsy was performed to assess pre-symptomatic Gb3 deposition, which was absent. Subsequently, cardiac MRI showed normal ventricular volumes, systolic function and wall thickness but slightly reduced native myocardial T1 values.

**Fig. 1:**
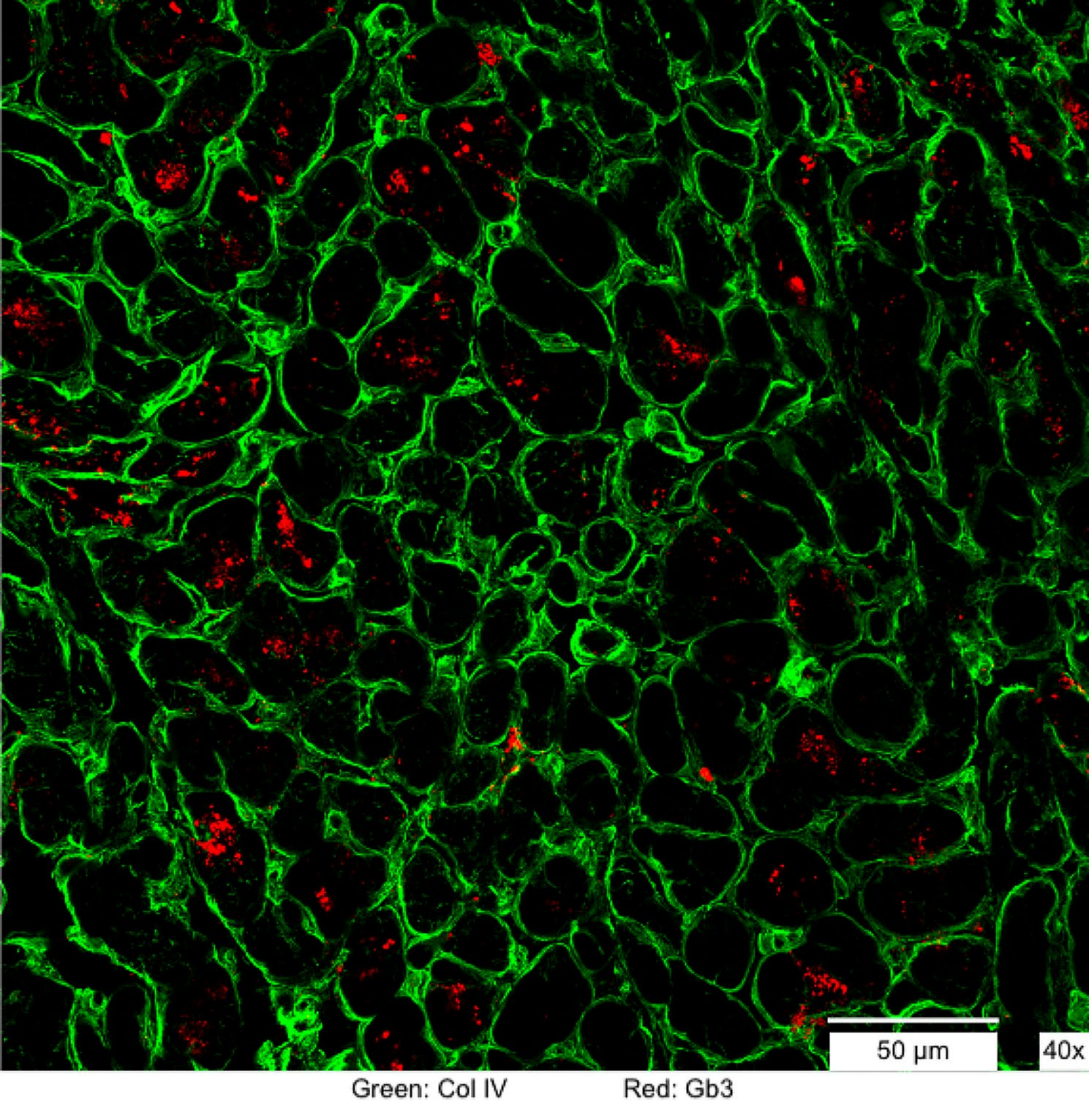
Cardiac biopsy showing type IV collagen (green) and Gb3 (red) stains

On the basis of her father’s disease history and on suspicion of cardiac disease on a very early stage, endomyocardial biopsy was proposed to Patient 2 after careful evaluation of potential risks and benefits. The myocardial tissue showed mild myocytes enlargement and vacuolization at light microscopy and typical myelin bodies at ultrastructure. Additionally, cardiac tissue was analyzed by the same IF technique used for skin samples, revealing widespread Gb3 accumulations within the cardiomyocytes and irregular Gb3 distribution in the small vessels with only a few areas spared (Fig. 1).

ERT was started without side effects. Of note, cardiac MRI was repeated at 25 years of age but showed no variation compared to the previous one.

At the last cardiological evaluation at 26 years of age, physical examination, as well as echocardiogram and ECG, were normal. Furthermore, no neurological signs or symptoms have been reported to date.

## Discussion

This paper underlines three main points: (1) the atypical clinical presentation of a classic *GLA* mutation, (2) the diagnostic usefulness of endomyocardial biopsy and (3) difficulties in the management of asymptomatic female carriers.

The *GLA* mutation presented in this paper (W162C) is reported as ‘classic’ in the literature, however information about clinical presentation is scant. It was first described in 1996 [1] in a 22-year-old female with proteinuria/hematuria and more recently in a 49-year-old male with severe hypertrophic cardiomyopathy, heart failure and mild renal impairment [2]. This mutation was determined to affect an amino acid residue of the α-galactosidase A “buried” in the hydrophobic core, thus altering protein folding and function [3, 4] and is listed among the non-amenable mutations for oral chaperone treatment. In our clinical case the proband, despite undetectable levels of enzyme activity, exhibited a later-onset phenotype with dominant cardiac involvement, associated with renal and brain abnormalities, without clinical signs of the classic form (cornea verticillata, angiokeratoma, neuropathic pain). Accordingly, skin biopsy resulted negative, confirming the already known aspect that a strict genotype-phenotype correlation is not present in FD [5].

Review of the literature concerning the mutation is summarized in the following table (Table 1):

**Table 1:** Review of the literature concerning the W162C mutation of *GLA*

Authors (year)	Key features
Germain et al. (1996) [1]	First description, female carrier
Garman et al. (2002) [3]	Mutation localized in hydrophobic core of GLA
Saito et al. (2013) [4]	Confirmed findings of [3]
Xiao et al. (2019) [6]	Study of microRNA expression, one W162C patient with classical disease

In female patients, the skewed inactivation hypothesis concerning the X chromosome has been recently tested by Wagenhäuser et al. [7] in a series of patients, including an asymptomatic woman carrying a W162C mutation: actually, no skewed activation was demonstrated in this individual (nonetheless, the study showed that X-inactivation patterns couldn’t predict disease phenotype).

The second issue highlighted in this paper is the importance of endomyocardial biopsy to disclose abnormal sphingolipid accumulation and allow early diagnosis in the presence of subtle clinical changes. Namely, biopsy showed overt abnormalities while cardiac MRI was unremarkable except for a slight native T1 values lowering in keeping with an initial lipid storage.

Additionally, this case underlines the challenge in the management of asymptomatic female carriers in FD. It is known that FD clinical course in this scenario is difficult to predict for several reason: type of gene mutation, lyonization profile, genetic and epigenetic background. Furthermore, ERT may not be appropriate in asymptomatic heterozygous female patients, with guidelines suggesting regularly multidisciplinary monitoring to promptly detect early disease presentation. In this perspective, cardiac MRI has demonstrated its significance as a crucial diagnostic tool in FD by detecting Gb3 storage through low native myocardial T1 values. Particularly, cardiac MRI is valuable in FD patients with normal wall thickness, as low T1 values can be observed in half of the patients without LVH, when a progressive subclinical myocyte storage occurs.

Current evidence points to Gb3 in tissues being a driver of inflammation, organ disfunction and thus disease [9]. While complications from tissue biopsy are possible (as in all invasive procedures), a risk-benefit ratio should always guide clinical decisions and we believe that in cases such as those similar to Patient 2’s the benefit to improve the outcomes outweighs the potential risks. Of course, the patient has the final say and should decide after complete elucidation of his/her situation. Thus, in individuals with due suspicion or affected relatives, “aggressive” evaluation in order to provide early diagnosis and disease-modifying therapy is perhaps justified.

## Conclusions

Fabry disease is a complex condition where natural history is difficult to predict based solely on gene mutation. This paper presents a late-onset clinical presentation with dominant cardiac involvement of a *GLA* mutation known as classic form mutation. Additionally, we underline here the role of advanced techniques for cardiac assessment in asymptomatic carrier, being able to detect glycosphingolipid storage before LVH is evident.

## Data Availability

All data generated or analysed during this study are included in this published article.
